# Padmabhushan Sri. Vaidhyabhushanam K. Raghavan Thirumulpad - an exemplary physician and guru

**DOI:** 10.1016/j.jaim.2025.101217

**Published:** 2025-11-05

**Authors:** Nikhila Sankar M, Jyotsna Govindan, Aswin T. Das

**Affiliations:** a Dept of Kriyasareera, Ashtamgam Ayurveda Chikitsalayam and Vidyapeedham, Vavanoor, Koottanad, Palakkad, Kerala, 679533, India; b Dept of Salyatantra, Ashtamgam Ayurveda Chikitsalayam and Vidyapeedham, Vavanoor, Koottanad, Palakkad, Kerala, 679533, India; c Dept of Salakyatantra, Ashtamgam Ayurveda Chikitsalayam and Vidyapeedham, Vavanoor, Koottanad, Palakkad, Kerala, 679533, India

The Fig. 1 understanding the life of historicalyesteryear's luminaries is necessary to provide inspiration to those who follow their path. Sri. Vaidyabhushanam K. Raghavan Thirumulpadu was such a beacon of Ayurveda, whose wisdom comes through his disciples and his works. Though generally known as a doyen in Ayurvedic science, his versatility was limitless and his proficiency extended beyond the bounds of Ayurveda. An excellent and empathetic physician, an insightful writer, a perceptive poet, a philosopher who absorbed the essence of different philosophies and incorporated them in his life, knowledgeable in *Tarka*, *Vyakarana* and *Jyotisha*, a teacher who became a refuge to students seeking pure Ayurveda, a Gandhian who lived his words, a Sanskrit scholar well-versed in all major classics including Ayurvedic treatises, a genius author who has numerous books on diverse topics to his credit; the list goes on (see Table 1).

**Fig. 1:**
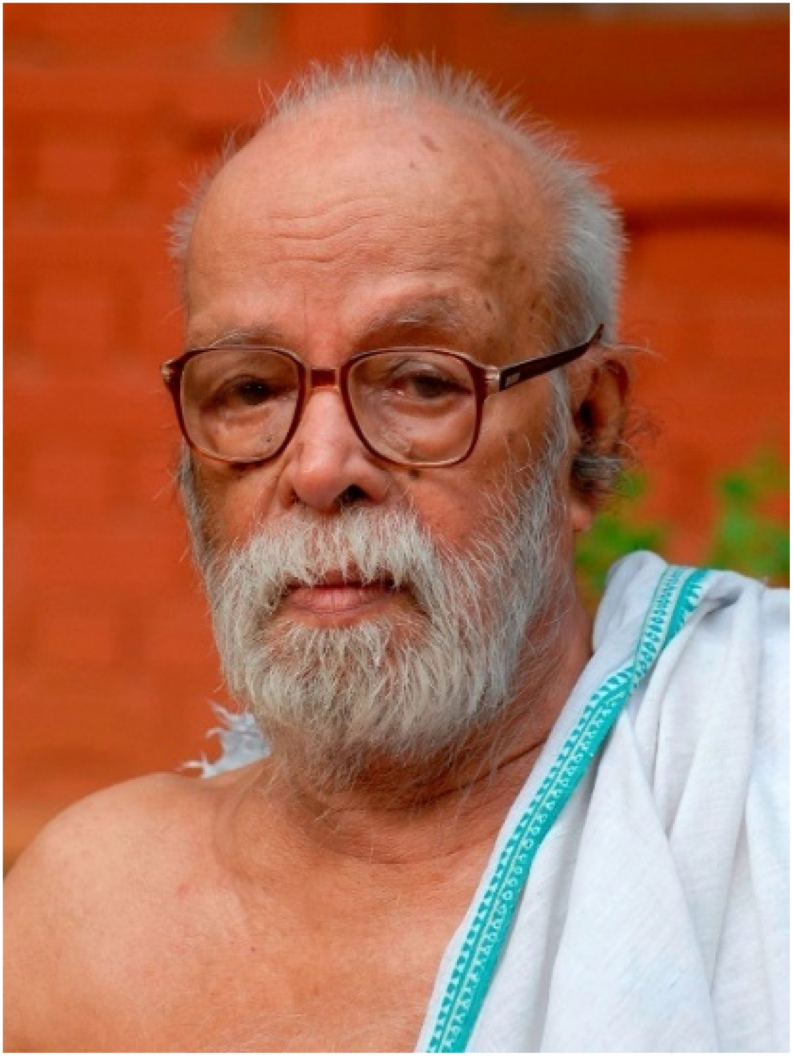

**Table 1:** Works of Sri. Vaidyabhusanam Raghavan Thirumulpad.

Sl. No.	Name	Year of publication	Details
1.	Bhagavatgita	1954	A verse by verse Malayalam transition of Bhagavatgita based on Sankarabhashya
2.	Prakriti chikitsa	1957	Incorporation of the knowledge he attained from Ayurveda, Naturopathy and from Gandhiji's book, KEY TO HEALTH.
3.	Buddhadarma	1958	Malayalam versification of Dharmapada, which had an introduction by Dalai Lama. An in depth study in buddhism.
4.	Devi Mahatmyam	1960	Malayalam versification of Sanskrit hymn of Goddess.
5.	IsavumMahatmaavam	1961	A treatise on Gandhian philosophy and strong critical reply to the Marxist criticism on Gandhism.
6.	Kriyakramam	1963	On the techniques of special Ayurvedic treatments of Kerala.
7.	Raghaveeyam	1972	Sthothrakavya on Lord Rama in Sanskrit.
8.	Hridroga (co- author)	1973	Ayurvedic approach on heart diseases
9.	Ayurveda Parichayam	1976	A comprehensible introduction to Ayurveda
10.	Thanthrayukti Vivekam	1976	A commentary on Tantrayukti Vichara by Neelamekhabhishak
11.	Rasavaiseshikavyakhya	1977	A commentary on Rasavaisheshika Sutra.
12.	Mukhakkannadi	1982	An autobiography published, when he reached the age of 60 (Shashtipoorthi)
13.	Vikritivyakhya	1982	Commentary on the portion of basic principles in Ashtangahridhaya.
14.	Ayurveda Darsanam	1983	Outline of Ayurvedic approach to health and disease.
15.	Ashtanga Samgraham series	1981–1987	Translation of Astanga Samgraha by Acharya Vagbhata into Malayalam along with commentary named PRAKASIKA, in 12 vol
16.	Ashtanga Darsanam	1998	About Ashtangas of Ayurveda – awarded by Kerala State council for science technology & environment
17.	Bhaishajya Darsanam	2002	(Articles on Ayurveda) – received G.N Pilla endowment award from Kerala Sahithya Academy
18.	Vicharadhara- 1& 2	2005, 2007	

## 1 Early life and education

Born on 20^th^ May 1920 at Chingoli, Alappuzha district, to Smt. K. Lakshmikutty Nambishtathiri and Sri. D. Narayan Iyer, he was, the eldest among their six children. Poor financial condition of his family was never an obstacle for him as he excelled in academics at school, and won scholarships which boosted his studies. But after completion of high school in 1937, from Government high school, Chalakudy; he had to give up the idea of collegiate education, due to lack of financial resources. But informal education continued and he learned Kalidasa's *Maagham* from one of his relatives, while residing at his father's hometown, Thirunelveli. Once he returned, he learnt *Jyothisham* (Astrology) under Sri. Mandaparampathu Narayanan Namboothiri. However, he had to leave it unfinished due to his master's poor health. It was at this time, that he had an encounter with the famous Sanskrit scholar and teacher at Chalakudy convent high school, Sri. Chendamangalam Ayyasastrikal, under whomhe got an opportunity to learn *Koumudi* and was fortunate to become one of the favorite disciples. This bond with his guru was so deep that even after becoming a busy Ayurvedic practitioner, he accompanied and served him in his last days, at his guru's behest.

After establishing a thorough base in Koumudi, he diverted his interest towards *Tarka* (Indian Philosophy) which he learned under his high school teacher, Sri. Konath Krishna Warrier, who was the disciple of Sri. Pareekshith Thamburaan. He also underwent training in Sanskrit under Sri. P. S. Subbarama Pattar, then Professor at Sri Kerala Varma College, Thrissur. His studies were interrupted, as he was appointed in Indian Railways department, in Chennai (then Madras) in the 1940s [1].

## 2 Life changing moment-towards the path of Ayurveda

The job in the railways had a big toll on his health, as within one year, he was diagnosed with Tuberculosis. He soon had to resign the job in railways due to poor health. Ironically, a curse became a blessing and it was this unfortunate event that directed him towards Ayurveda. His sickness was treated by Ayurvedic management in the hands of Vaidya P. Vasudevan Nambisan, chief physician, Rama Varma Central Ayurveda Hospital, Thrissur. This sparked his interest in Ayurvedic science and under the guidance of this same physician, Sri. Thirumulpad learnt Ayurveda in the traditional *Gurukula* system method, during 1944–1949.

In accordance with the *Gurukula* method, he stayed at the *Guru's* residence and served the *Guru* obediently and dedicatedly learned the science for five years. On 5^th^ April 1950, he was conferred Vaidyabhushanam Degree from the Government of Kochi, after completion of the course. He did internship in the same hospital, with *Panchakarma* as specialty for a year and got A-class medical registration from Travancore-Cochin Medical Council.

During 1950s- 60s he was widely influenced by the ideas of Mahathma Gandhi and became an ardent Gandhi follower. This motivated him to promote Naturopathy, use of Khadi, propagation of Hindi language, and Sarvodaya. He followed the example of the legendary physician Dr. B. C. Roy and engaged in social service every evening, after his clinical practice. He has also written several articles based on Gandhian views. He got a chance to learn Naturopathy for one month from Indian Institute of Natural Therapeutics, Bombay and translated an English speech of a Naturopathy expert to Malayalam [2].

## 3 Personal and family life

Sri Thirumulpad married Ms. Vishalakshi Thampuratty and they have four sons Dr Murali, Sri. Mukundan, Sri. Murari, Sri. Ravi Varma and a daughter Dr. Muthulakshmy. Dr Murali followed his footsteps to become a well-known Ayurveda physician and teacher. His daughter Dr. Muthulakshmy is a Sanskrit Professor, currently the pro vice-chancellor of Sree Sankara University of Sanskrit, Kalady. His wife predeceased him in January 2009.

## 4 Knowledge tradition - a vision

Ayurvedic education system changed hugely in the 1990s, and collegiate system was formally established as the authentic method, which was completely different from the former *Gurukula* system. Sri Thirumulpad had a welcoming attitude to this change of approach and used to say “I teach in the style I am used to, my students practice it in the way they are comfortable with. I see no problem in this arrangement". Despite the establishment of the collegiate system, students from different colleges of Kerala sought out his guidance and were regular followers at his hospital. By 1990s his name was known all over Kerala, and he was considered by many as a modern day Acharya.

He, however, has always stood against the commercialization of medicine and has never insisted on fees from the lakhs of patients he treated. Many young Ayurvedic practitioners and students, who became his disciples, practiced his way of treatment which is distinctive with less use of medicines, a strict following of *Pathya* and *Nidanaparivarjanam*, by lifestyle modifications. His way of teaching was also peculiar, which enriched the students with a relearning of the *Samhitha*, especially Ashtanga Hridaya based on *Darshanas* and a clear understanding of the Sanskrit verses. The more receptive disciples were also trained in Sanskrit, through study of Sanskrit literature. The excellence of his teaching is evident in the brilliance of his disciples, who later became prominent figures in the Ayurvedic community of Kerala [2].

## 5 Literary contributions

His professional career and literary prowess must have developed concurrently, since his earliest literary works are dated back to the1950s, soon after he acquired his degree. His writings range through translations, commentaries, critical reviews, articles, essays, poetry, and reviews in varied subjects like Sanskrit, Philosophy and even devotional books along with Ayurveda. The essence of his versatility stems from the variety and range of expert education he received as a student.

Another feather in his cap is that he had participated in the essay writing competitions conducted by Kottakkal Arya Vaidya Sala for 15 years, out of which he was one among the finalist for 13 years consecutively and bagged first prize 8 times. These essays were published every year by the Arya Vaidya Sala. His knowledge in Sanskrit, *Vyakarana* and *Tarka* helped him to understand Ayurveda in its depth and to write commentary for Ashtanga Sangraha called Rasavaisheshikam. He also taught Sanskrit for Sanskrit Vidvan examination.

He held major positions on the academic front in all of the main universities in Kerala. He was a Board of Studies member in M. G. University & Calicut University as well as Faculty of Ayurveda in Kerala University & Kannur University. He was also a Syndicate member and Academic council member in Sree Sankaracharya Sanskrit University. He also gave guidance to PhD students in Sanskrit and Ayurveda. Dr. M. S. Valiathan (Founder director of Sree Chitra Institute of Medical Sciences) had sought his opinion while writing “the Legacy of Caraka” – a study based on Caraka Samhita. He was also an illuminating orator on social, scientific and theological matters. Apart from this, he has also participated in presentations and discussions inside and outside Kerala [3].

## 6 Establishment of VKRT foundation

In 1970, he founded the Ayurvedic Medical Practitioners Hospital and Industrial Co-operative society Ltd. (AMPHIC) pharmacy, a co-operative society of Ayurvedic physicians running a manufacturing unit of Ayurvedic medicine of superior quality at reasonable price. He also laid the foundation, in 2000, for Vaidyabhushanam K. Raghava Thirumulpad Foundation of Ayurvedic Studies for propagation of classical Ayurveda through publication of books, organizing seminars and classes and conducting annual thesis competitions for students of Ayurveda and Sanskrit.

Similar to how Sri. Thirumulpad used to visit his Guru's house accompanied by his son Dr. Murali, as well as his disciples like Dr. M. Prasad and many others; it was hearty to see his own disciples following him till his demise. His devotion to his masters were repaid with the devotion of his pupils. On the occasion of his 90th birthday on 23^rd^May 2010, they released 9 of his books as part of ‘Navathi Pranamam’ by their committed effort.

Two months following his death, on 21^st^November 2010, at the age 90 years, the great scholar was posthumously awarded with Padma Bhushan, on 25^th^ January 2011. India Post dedicated a stamp with the image of Sri K. Raghavan Thirumulpad as a part of its theme Master Healers of AYUSH, and was released by the Prime Minister, Sri. Narendra Modi, on 30th of August 2019 [4].

He is also the recipient of various other awards, out of which some of the major ones include:

1. Acharya, Ayurvedaacharya, Ayurvedavachaspathi awards by Samskrutha Sabha
2. Pandita Ratna award by Vishva Samskruta Prathishtan
3. Vidyabhushanam Award by Kerala Sanskrit Academy
4. Fellowship from National Academy of Ayurveda, Delhi
5. Bhishak Parama Acharya award by Nagarjuna Research Foundation
6 Kerala Pathanjali award by All India Ayurveda Medicine Manufacturers Organisation
7. Ashtanga Ratna award by Government of Kerala.

His goal of comprehensive learning and propagation of Ayurveda, still finds reality through the Vaidyabhushanam K. Raghavan Thirumulpad foundation for Ayurvedic Studies. The foundation actively works towards these goals with its initiatives like conducting seminars, holding discussions, Vagbhata Sameeksha (closed door creative discussion on classical textual contexts), Swadhyaya (self-study of classical text), and Vaidyasamvada. Vaidyasamvada is a trimonthly published by the trust, which includes scientific articles written by him [2].

Sri Vaidyabhushanam K RaghavanThirumulpadwas a great Ayurvedic scholar who contributed greatly to the propagation and development of Ayurveda in Kerala. His published literary works have ever since been the reference texts for Ayurvedic students in Kerala. He was an excellent teacher, a great writer and orator. He absorbed Ayurveda in its true sense while also imbibing the philosophies of other contiguous branches of medical sciences like Naturopathy. He was, and is an inspiration for all who work for Ayurveda and intend to study and practice it earnestly.

## 7 Future of Ayurveda through the eyes of the legend

In the recent times of increased Global recognition and commercialization of Ayurveda, the ideologies and wisdom of Vaidyabhushanam K Raghavan Thirumulpad could become the guidelines in the preservation of pure Ayurveda.

• Minimalization of medicines to prevent Aushada Ajeerna in a patient and thus protecting his Agni.
• Controlled utilization of medicinal plant resources.
• Propagation of Shamana chikitsa over Shodhana, especially in the climatic conditions of Kerala, needs to be highlighted.
• Relying on Pathya and Apathya knowledge rather than Aushada prayoga helps in maintaining health without undue use of medicines.
• Advocating and implementing Dinacharya and Ritucharya in day to day life prevents many diseases.
• According to his view, Vaidyas have the responsibility of showcasing (acharana) the Ayurvedic method of life to the society.

His greatness lies in the fact that his inquisitive mind never stopped learning and was ever eager to pass on that knowledge to the next generation. Though his physical body is no more, he will live through the treasure of knowledge he left behind and through the countless lives he has touched by virtue of his selfless contributions to the society.

## Author contributions

NS: Conceptualisation and documentation, JG: Editing and revising, AD: Data collection and sorting.

## Declaration of generative AI in scientific writing

We hereby declare that none of the types of generative AI was used in the writing of this article.

## Sources of funding

None.

## Conflicts of interest

None.
